# An East Meets West Approach to the Understanding of Emotion Dysregulation in Depression: From Perspective to Scientific Evidence

**DOI:** 10.3389/fpsyg.2019.00574

**Published:** 2019-03-28

**Authors:** Jiajia Ye, Shuhe Cai, Wai Ming Cheung, Hector W. H. Tsang

**Affiliations:** ^1^ Department of Rehabilitation Assessment, Fujian University of Traditional Chinese Medicine Subsidiary Rehabilitation Hospital, Fuzhou, China; ^2^ Department of Rehabilitation Sciences, The Hong Kong Polytechnic University, Hung Hom, Hong Kong, China; ^3^ Department of Orthopaedic Rehabilitation, Fujian University of Traditional Chinese Medicine Subsidiary Rehabilitation Hospital, Fuzhou, China; ^4^ Faculty of Education, The University of Hong Kong, Hong Kong, China

**Keywords:** traditional Chinese medicine, depression, East meets West, neuroscience, neurophysiological pathway, emotion

## Abstract

Depression, an emotion regulation disorder, is a prevalent mental illness in the world. Meanwhile, traditional Chinese medicine (TCM) has been increasingly regarded as a promising and effective alternative therapy approach for patients with depression. Despite many years of research on depression, the current understanding of the pathological mechanism of depression based on TCM theories is still in its infancy. Due to the lack of scientific evidence in the past, TCM is not fully recognized by researchers around the world. This review firstly summarizes the pathogenesis and etiology of depression in terms of both Eastern and Western medical systems. Secondly, it adopts an integrated Eastern and Western approach to propose some plausible neurophysiological pathways linking the liver, spleen, and heart functions explicated in TCM theory. The aim of this theoretical review is to bridge the knowledge gap between Eastern and Western medicine, which may better explain the pathology of depression.

## Introduction

Depression, an emotion regulation disorder, is one of the most prevalent psychiatric disorders worldwide. This disorder will become the second leading cause of disability by 2020. People with depression will spend approximately 8.2% of their lifespan struggling with the associated disabilities (Ferrari et al., 2013). The prevalence of depression in adolescents is high, accounting for 6% of the population. Recent epidemiological studies indicate that the lifetime rate of depression is 16% (Andrade et al., 2003; Kessler et al., 2003). Depression includes such symptoms as fatigue, depressed mood and anhedonia, irritability, loss of appetite, body weight changes, and sleep disorders. These symptoms may lead to a heavy burden on the patients, their families, their friends, and society (American Psychiatric Association, 2013).

Emotion regulation refers to the interaction between the occurrence, intensity, duration, and expression of emotion (Gratz and Roemer, 2004). It is widely acknowledged that emotion regulation strategies are closely associated with mental health (Aldao et al., 2010). Depression is characterized by the emotion of sadness and the inability to extract pleasure from positive situations. Previous studies have suggested that patients with depression lack the emotion of anger because of their inability to handle stressful situations (Gu et al., 2016, 2018a). There are many ways for people with depression to regulate their emotions, including coping strategies and motivation (Kring and Werner, 2004; Campbell-Sills et al., 2006). A number of experimental studies on emotion regulation support the view that deficit in emotion regulation can be a crucial clue to understanding the etiology of depression (Soygüt and Savaşir, 2001). Therefore, emotion dysregulation is closely related to depression, and it is vital to understand emotion regulation in order to unravel the pathogenesis of this disorder.

Traditional Chinese medicine (TCM) originated from ancient China and has evolved over thousands of years. Nowadays, a growing number of people around the world are using TCM to prevent or cure diseases. In 2006, there were over 200 million outpatients and 7 million inpatients receiving TCM therapies. Most of the principles of TCM are derived from the philosophical basis of Taoism and Confucianism (General Office of the State Administration of Traditional Chinese Medicine and School of Management of Beijing University of Chinese Medicine, 2006). The main TCM therapies include herbal medicine, acupuncture, acupressure, moxibustion, massage, cupping, and physical exercise such as qigong. TCM theory is based on clinical experience instead of scientific evidence. Western medicine, on the other hand, is based on scientific investigations and tested by animal experiments and clinical trials. The two systems differ in their diagnoses, treatments, and theories (Tian, 2011). Despite a long history of clinical experience, the fundamentals of TCM remain largely unchanged and, similarly and unfortunately, the scientific elements underlying its theories remain largely unknown (Keji and Hao, 2003). Lack of scientific evidence has led to skepticism, criticism, and even rejection of TCM (Ted, 2000).

Given the high prevalence of depression and the increasing attention given to TCM, this theoretical review attempts to explore the etiological mechanism of depression *via* the Eastern and Western or integrative approach. In the long run, this paper will broaden and deepen our understanding of the etiology, signs, and symptoms of depression. Hopefully, this will give us insight into the development of innovative intervention strategies.

## Traditional Chinese Medicine’s Views About Emotion and Depression

The TCM theory of emotion has a history of more than 2000 years and embraces well-established diagnosis and treatment systems. Many ancient Chinese texts have contents pertaining to the syndromes, etiologies, and treatment of depression caused by extreme emotional changes using the concept of “yu” or “yu-zheng,” which literally means “not flowing, entangled, blocked, or clogged” (Ou, 1988). *The Yellow Emperor’s Classic of Internal Medicine* is usually considered the earliest Chinese classic medical text in the world (General Office of the State Administration of Traditional Chinese Medicine and School of Management of Beijing University of Chinese Medicine, 2006). It expounds the relationship between emotional changes and the five viscera, namely, the heart, spleen, kidney, liver, and lung. According to TCM theory, emotional change is closely related to the etiology of diseases. The five viscera parallel the five elements (i.e., metal, wood, water, fire, and earth) which are transformed to create joy, anger, sadness, missing, and fear (Veith, 2015). This is derived from the five elements theory which can be used to understand the physiology and pathology of the human body and the etiology and pathogenesis of diseases (Giovanni, 1989). The transformation of emotion is based on the productive cycle of the five elements theory. The interaction of elements and organs is as follows: sadness is related to the lung, and joy can oppose it; fear is related to the kidney, and missing can oppose it; anger is related to the liver, and sadness can oppose it; joy is related to the heart, and fear can oppose it; missing is related to the spleen, and anger can oppose it (Giovanni, 1989; Chen, 1990; Gu et al., 2018a,b). Theoretically, emotional changes have two-way functions. On the one hand, emotional changes may lead to specific diseases. On the other hand, some diseases may result in emotional changes. If emotional changes (i.e., anger, fear, and sadness) can be managed in the short term, this would not bring about negative influences on the human body (Wang et al., 2017). If emotional changes are strong and last for a long time, this will give rise to the dysregulation of the autonomic nervous system (ANS) because it exceeds the adjustable range of physiology and depression will occur.

**Figure 1:**
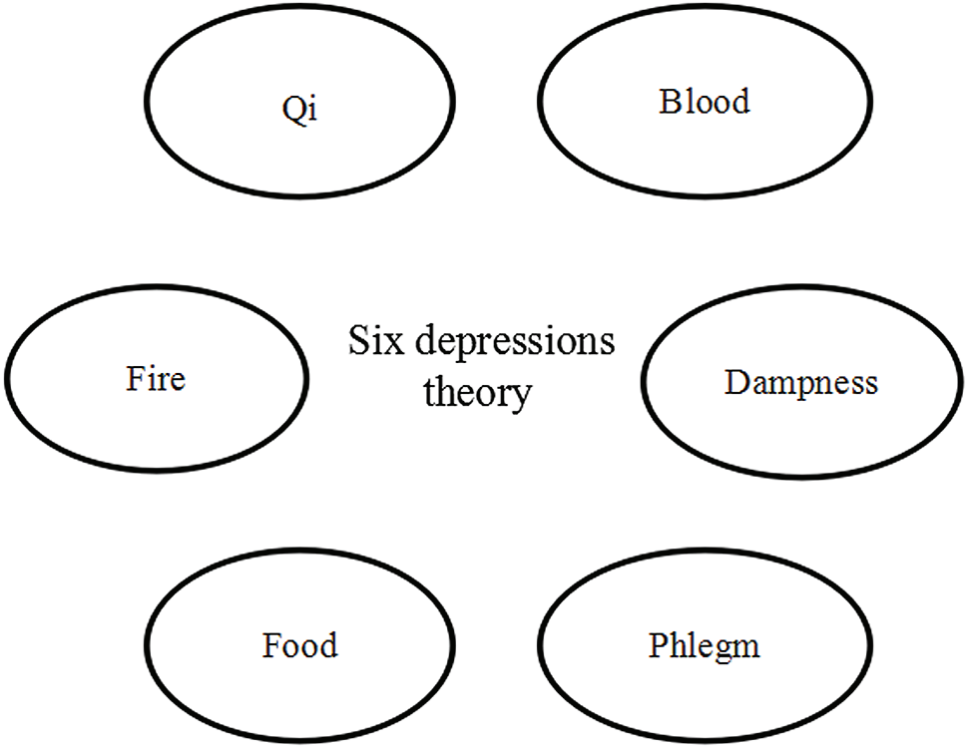
Traditional Chinese medicine theory.

Many ancient Chinese practitioners proposed definitions of yu. Tao Hongjing (Wu et al., 1963), the author of *Shennong Bencao Jing Jizhu*, a variorum of Shennong’s classic materia medica, and a physician of the North and South kingdoms period, reported the treatment of yu using antelope horn. In another text, Chen Wuzhe (Chen and Lu, 1995), who was a famous TCM practitioner in the Song dynasty (960–1,279), proposed the concept of the “seven emotions” which indicated that emotional changes may lead to disharmony of the internal organs and then to yu. Zhang Congzhen (Zhang et al., 2011), who was the most famous TCM practitioner in the Jin dynasty (265–420), put forward the pathogen concept. Mr. Zhang proposed the methods of sweating, emesis, and diarrhea to treat the yu-zheng caused by a pathogen. Although several concepts regarding yu were proposed in ancient times, the most useful concept for understanding the progression of yu is Zhu Danxi’s theory of the six depressions, which is regarded as the mainstay of TCM theory for understanding depression. The theory of the six depressions involves the stagnation of either qi, blood, dampness, phlegm, food, or fire, and it is built on earlier Chinese medical texts such as the *Treatise on Cold Damage and Miscellaneous Disorders* and *The Yellow Emperor’s Classic of Internal Medicine* (Scheid, 2013; Chen et al., 2015). Zhu Danxi’s approach focused on the understanding of disease dynamics. He mentioned that qi was responsible for the movement and transformation of blood, dampness, phlegm, food, and fire. If the qi was stagnant, either blood, dampness, phlegm, or food would not be able to move or transform properly in the human body. These obstructions of substances might accumulate and eventually turn into fire (Park, 2002) (Figure 1).

When Western medicine was introduced into China, its nosology was usually translated into Chinese by referring to the closest TCM concept. In the case of depression, it was translated into Chinese as “yiyu” or “yu” with reference to the yu syndromes in TCM (Ng et al., 2006). Unlike Western medicine, in TCM, diagnosis is based on the syndrome differentiation of diseases or disorders underlying symptom co-occurrence patterns. TCM practitioners discover constraints not only by asking and observing but also by palpating and smelling (Ross, 1985).

The onset of depression is often due to significant emotional changes that are mainly related to the liver (Wang and Lu, 2002; So et al., 2015). In its initial stage, depressive syndrome is mostly classified as “excess type”; in prolonged cases, the classification changes to “deficiency type” or “deficiency-excess type” (Wang and Lu, 2002). Liver qi plays a vital role in the precipitation of depressive episodes, and stagnation of the liver qi is part of the excess-type classification. Conventionally, when there is emotional change, the liver qi is affected first, followed by disharmony of the qi among the five viscera, especially the liver, spleen, and heart, resulting in a loss of regulation of the qi and blood. If the liver fails to control the dispersion, the function of the spleen will be repressed. This will lead to dissipation and harm to the heart qi. If the heart loses its nourishment and the “shen” (spirit) becomes restless, it will lead to an unstable and depressed mood. When stagnation of the qi is prolonged, it will accumulate and transform into fire (Allen, 1990). Previous reviews have supported this theory and suggested that anger leads to deviant dispersion of the liver qi, which then causes depression (Zhao, 1992; Jin and Liang, 1997; Guo and Liu, 2002). A growing body of evidence suggests that anger may lead to liver dysfunction, which means that the liver’s function of spreading qi is impaired. Once the liver is unable to maintain its free and unobstructed flow, people may experience depression (Liu, 1991; Zhao and Zhao, 1999; Park, 2002).

## Western Medicine’s Views About Emotion and Depression

More and more studies are investigating the etiology of depression (Krystal et al., 2002; Smith and Vale, 2006; Moret and Briley, 2011; Liu et al., 2015). However, the underlying pathophysiology of depression is still not fully understood. Several possible theories may explain the potential process involved in depression, but neurophysiological factors play a vital causal role in the process (General, 2001).

### Regulation of Neurotransmitters

#### Norepinephrine Theory

Norepinephrine (NE) is responsible for the regulation of cardiovascular activity, pain sensation, and body temperature. Previous studies have shown that there is a close link between NE and anxiety (Schildkraut, 1965; Liu et al., 2015). The possible relationship between depression and disturbance of NE in the brain was first proposed in 1965 (Schildkraut, 1965). In an animal study (Schildkraut, 1965), it was reported that a lower concentration of NE in the brain caused by reserpine might lead to depression. Evidence showed that people with depression had either low or high levels of urinary 3-methoxy-4-hydroxyphenylglycol (MHPG), the metabolite of NE degradation, which indicated there are significant differences in the amount of NE in terms of synthesis and release between people with depression and healthy individuals (Samson et al., 1994). As noradrenergic pathways in the brain arise from the locus coeruleus and project to the frontal cortex, limbic system, and spinal cord, neuroimaging studies suggest that abnormal metabolism in the limbic and paralimbic structures of the prefrontal cortex (PFC) is associated with emotional dysregulation and depression, which might indicate that medicine that increases NE activity in the brain could be one of the most effective therapeutic agents (Drevets et al., 2002).

#### Serotonin (5-HT) Theory

Serotonin, biochemically derived from tryptophan, is primarily found in the central nervous system (CNS), the gastrointestinal tract, and blood platelets (Young, 2007). There are generally seven serotonin receptor subtypes which exert influences on various biological and neurological processes, such as aggression, anxiety, appetite, sleep, mood, and thermoregulation (Glennon and Dukat 1991; Wesolowska, 2002). Coppen et al. (1965) developed the hypothesis on 5-HT and the treatment of depression in 1965. They proposed that decreased levels of 5-HT in the synaptic cleft might result in depression. A study by Pandey (1997) found that suicidal patients had lower levels of 5-HT compared to normal subjects. A study by Wägner et al. (1990) showed that taking fluoxetine, a selective inhibitor of 5-HT uptake, significantly reduced the content of 5-HT compared to its original level based on a platelet sample and relieved the syndromes caused by depression. Clinical studies showed that 5-HT_2_ receptors were likely to be the candidates involved in the pathophysiology and treatment of depression among various 5-HT receptor subtypes (Hoyer et al., 1986; Nyberg et al., 1993). In addition to 5-HT_2_, 5-HT_1A_ receptors have an influence on the regulation of mood. A review suggested that the 5-HT_1A_ receptors were particularly related to antidepressant and anxiolytic responses in human beings (Blier and Ward, 2003). The presynaptic 5-HT_1A_ receptors are located in the raphe nuclei, where they act as cell body auto-receptors to inhibit the firing rate of 5-HT neurons. On the other hand, the postsynaptic 5-HT_1A_ receptors are located in the limbic and cortical regions, where they also attenuate firing activity, which indicates that 5-HT_1A_ receptors bring about a negative feedback influence on firing activity in the brain (Aghajanian and Lakoski, 1984; Blier and De Montigny, 1987; Blier and Ward, 2003).

### Dopamine Theory

Dopamine (DA), which participates in emotion regulation, is produced by the dopaminergic neurons in the ventral tegmental area (VTA) of the midbrain, the substantia nigra pars compacta, and the arcuate nucleus of the hypothalamus, and its notable functions are associated with the mediation of mood, behavior, and cognition (Martini, 2015). The relationship between DA and depression was first developed by Molander and Randrup (1976). Willner (1983) found that the concentration of DA was lower in patients with depression compared to healthy subjects. A study with post-mortem human subjects showed that the metabolite rate of DA was critically decreased in suicidal patients with depression, specifically in the regions of caudate, putamen, and nucleus accumbens (Bowden et al., 1997). Evidence from recent studies also supports this finding. An animal study showed that depletion of DA in brain samples was found in animals with behavioral depression after 3 weeks of reserpine injections (Ikram and Haleem, 2017). A clinical study found that the D_2_ receptor of DA might be supersensitive in patients with depression compared to controls by means of a novel neuroendocrine challenge test which indicated that dopaminergic mechanisms might be a target of therapeutic interest (Verbeeck et al., 2001).

### The Relationship of Possible Factors

#### Glutamine and λ-Amino Butyric Acid

Glutamine (Glu) and λ-amino butyric acid (GABA) are respectively the main excitatory and inhibitory amino acids in the CNS mediating general mood states (Crabtree et al., 2013). Increasing evidence from clinical studies shows that Glu levels decrease in depressed patients compared with healthy controls (Auer et al., 2000; Liu et al., 2015) and GABA concentrations in the occipital cortex and prefrontal regions of patients with depression also decrease compared with control groups (Sanacora et al., 1999; Hasler et al., 2007). Studies on TCM have mentioned that levels of Glu and GABA might be increased through taking Chinese herbs (Gao et al., 2014; Liu et al., 2015). As the levels of Glu and GABA are vital to maintaining normal brain function, the two neurotransmitter systems may be the possible therapeutic targets in depression (Zorumski et al., 2013).

#### Gene and Environment Interaction

Previous research on twins has demonstrated that genetic factors play a vital role in the development of depression. Scientific findings show that the heritability of depression accounts for between 31 and 42% of the variance in adolescents’ depressive symptoms (Sullivan et al., 2000; Barclay et al., 2015). Scientists have recently raised the possibility that genetic vulnerability factors can interact with environmental factors to make depressive symptoms more severe. An empirical study has suggested that social context will have a function in triggering and compensating for a genetic diathesis (Heath et al., 2002). Also, social context will act as a control to prevent “genetic predisposition behaviors” (Heath et al., 2002). The diathesis-stress process of the gene-environment (GE) interaction might occur when those who have genetic vulnerability are under a stressful environment (Shanahan and Hofer, 2005).

#### Cognition

**Figure 2:**
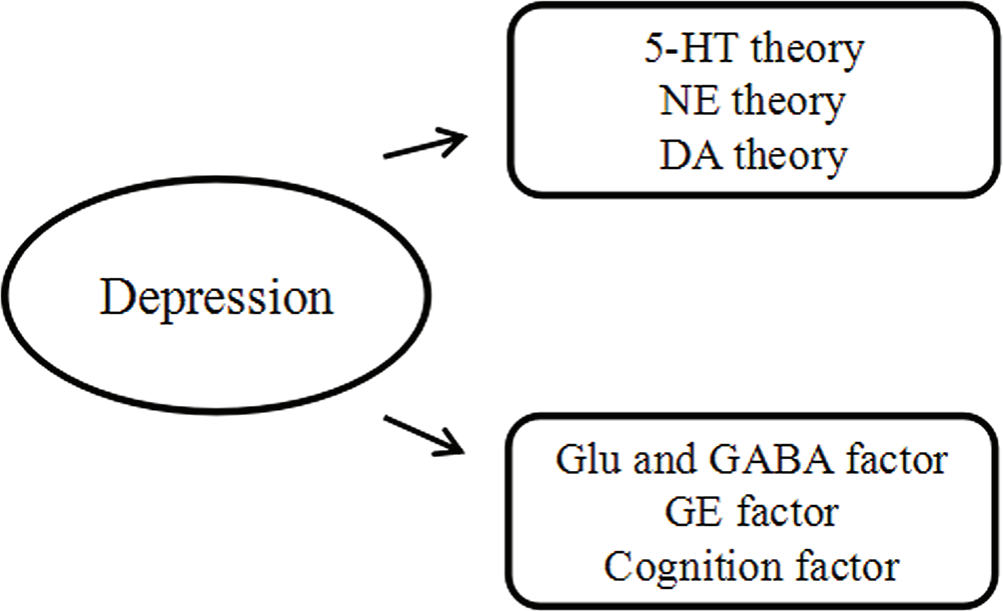
Western medicine theory.

Cognition refers to the mental actions or processes of (a) gaining new knowledge and understanding and (b) recalling memories that involve perceiving, recognizing, conceiving, and reasoning. Apart from the factors of neurotransmitters and GE interaction, Beck’s cognitive theory of depression also has to be taken into consideration. The cognitive theory of negative automatic thoughts and underlying dysfunctional assumptions schemas were proposed by Beck in the mid-1960s (Beck, 1979). He found that the negative way of thinking came from previously unpleasurable experiences which could guide people’s perceptions or interpretations, hence leading to a negative worldview and causing depression (Soygüt and Savaşir, 2001). The cognitive theory of depression indicates that early relevant experiences might result in the formation of dysfunctional beliefs which might lead to negative self-beliefs. When those who have negative self-views about themselves encounter a specific circumstance, they are more likely to feel hopeless and useless and ultimately be depressed (Soygüt and Savaşir, 2001). A study by Allen (1990) based on students showed that negative attitudes toward the future was related to depressive mood, and depression-prone students were found to negatively process personal information, leading to the development of symptoms of depression. Evidence from Abela and D’Alessandro (2002) was in line with previous findings and suggested that dysfunctional attitudes and an increase in depressive mood were significantly associated with students’ negative beliefs about the future (Figure 2).

## An Integrated East Meets West Approach to Closing the Gap

In TCM, “zang fu” can be translated as “internal organs.” It may be regarded as a core concept of TCM which views the physical body as an integrated whole. It also describes an integrated relationship between mental activities, sense organs, tissues, five solid and six hollow organs, and environment influences (Giovanni, 1989).

The theory of internal organs is entirely different from the anatomical structure originating from Western medicine. However, this does not mean that TCM entirely disregards anatomy. The concept of organs in Western medicine is based on anatomy, whereas the concept of organs in TCM is based on a system concept that embraces anatomy, physiology, and psychology. In TCM, the function of internal organs is basically related to various substances, emotions, tissues, and senses. For example, the basic substances of TCM are qi (energy), xue (blood), jing (essence), shen (spirit), and jin ye (body fluids). Each substance is related to one or more organs (e.g., the spleen governs food qi and influences body fluids, and the heart governs blood).

In Western medicine, the liver, the largest internal organ, has various functions in the body, including the synthesis of proteins, blood clotting factors, triglycerides, cholesterol, glycogen, and the production of bile. However, TCM theorists believe that the liver is responsible for controlling dispersion in all organs and in all directions to ensure the smooth flow of qi throughout the body. This is the most salient of all the liver’s functions, especially as far as depression is concerned. With reference to depression, the liver is postulated to be related to the functioning of the neuroendocrine system in Western medicine (Li and Wang, 1985; Yue and Tian, 1995).

To our knowledge, every organ’s energy has a normal direction of flow: the qi of some organs flows downward (such as that of the stomach) and the qi of other organs flows upward (such as that of the spleen). The normal direction of the movement of the liver qi is upward and outward in all directions to make sure that the flow of energy is smooth and unimpeded. There are three functional activities of the liver in terms of this movement: regulating emotions, regulating the secretion of bile, and assisting the digestive function of the spleen and stomach (Ross, 1985; Giovanni, 1989).

The emotional state of an individual in fact depends on the smooth flow of energy and blood. When the liver qi flows smoothly, the emotional status of the individual will be calm and peaceful. In contrast, if the liver is not functioning well, the energy of the liver will stagnate, which will then lead to an abnormal increase in liver qi, and give rise to emotional disturbances, such as depression, accompanied by physical symptoms, such as a sensation of oppression in the chest and hypochondriac pain (Giovanni, 1989). Scientific studies of animal and human subjects have provided preliminary support to the postulation that the liver function in TCM is associated with the neuroendocrine system that includes the regulation of the NE system located in the locus coeruleus (LC/NE) and the hypothalamic-pituitary-adrenal axis (HPA) (Yue and Tian, 1995; Wang and Yao 2002; Yan and Xu, 2005; Yue et al., 2005a).

### LC/NE System

**Figure 3:**
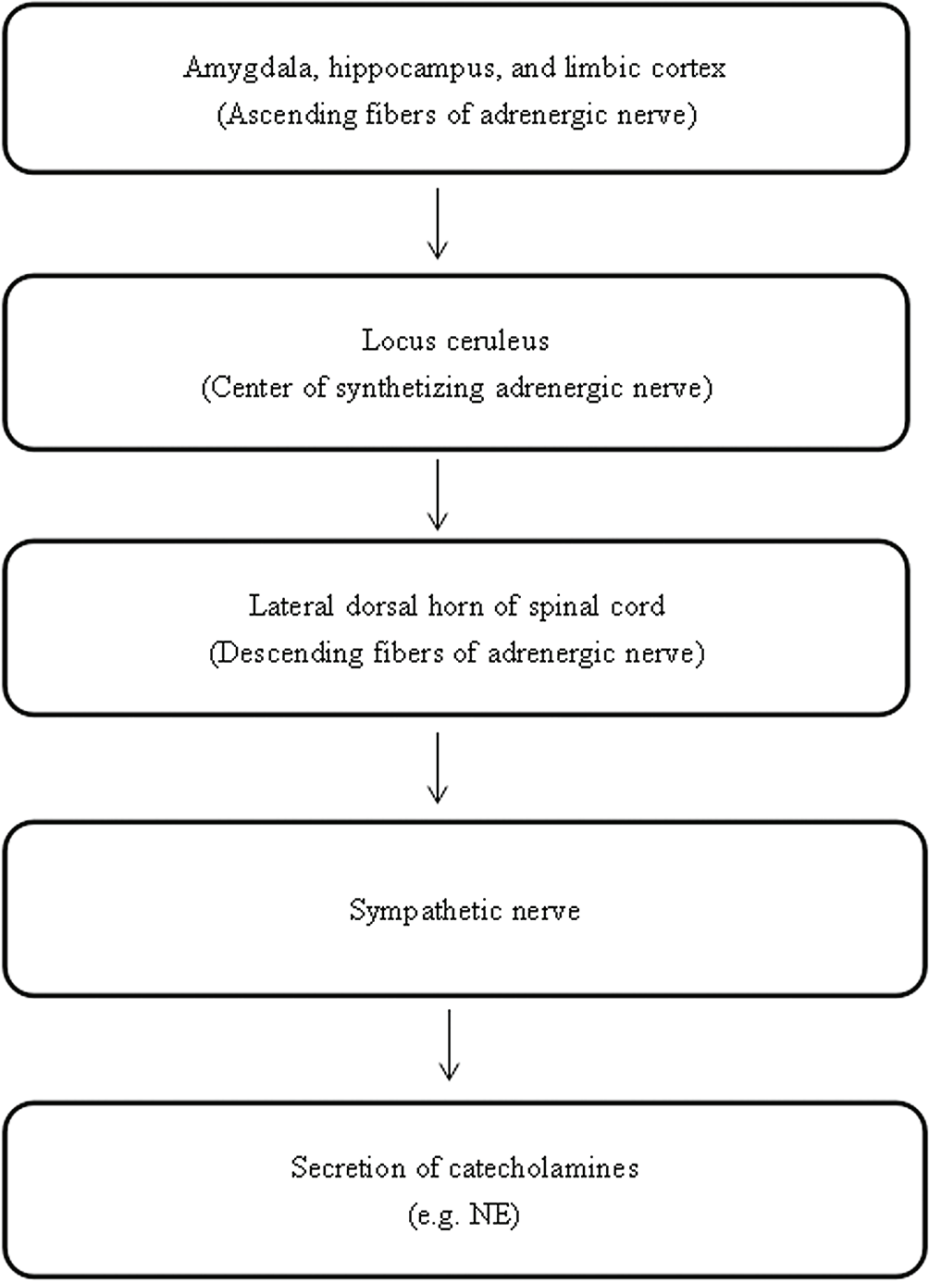
The neuropathways associated with the LC/NE system.

Studies have explored the symptoms of the abnormal rising of the liver qi that are correlated with a lack of regulation of the ANS (Yue and Tian, 1995), a deficiency of serotonin, and an excessive level of NE (Spiegelhalder et al., 2011; Wei et al., 2012). However, another study claimed that NE level is not related to the severity of depression because of the different stages of depression (Yuan et al., 2004). The LC/NE system may be involved in the regulation of the neuroendocrine system based on the syndrome of liver qi stagnation. The locus coeruleus is the central site of the LC/NE system in the brain, which is the center of the synthesizing adrenergic nerve. The ascending fibers of the adrenergic nerve are mainly projected into the amygdala, hippocampus, and limbic cortex, which are responsible for emotional changes, memory, and behavioral changes. The descending fibers of the adrenergic nerve are mainly projected into the lateral dorsal horn of the spinal cord, which is involved in the regulation of the activity of the sympathetic nerve, and the secretion of catecholamines. It has been suggested that the activated amygdala may stimulate the release of the corticotrophin-releasing hormone (CRH) that increases the activity of the sympathetic nerve *via* the mediating lateral dorsal horn of the spinal cord. Once the sympathetic nerve is activated, adrenaline medulla will release NE and epinephrine (E) due to the activated adrenal gland (Copstead and Banasik, 2010) (Figure 3).

A growing number of clinical trials support the association between liver diseases and the lack of ANS regulation (Yue et al., 2005a). A study by Jin (2000) mentioned that there is a positive correlation between increased sympathetic tone activity and the excess type of liver dysfunction, such as loss of appetite and wiry pulse, while there is a negative correlation between increased parasympathetic tone activity and the deficiency type of liver dysfunction, such as weak pulse. A study by Yuan et al. (2004) suggested that NE level is relatively higher in patients with depression compared to people in normal health. As the results on NE level in patients with depression are contradictory, experimental studies to explore this monoamine transmitter concentration in depressed patients would be a promising direction for further research.

### The HPA Axis

**Figure 4:**
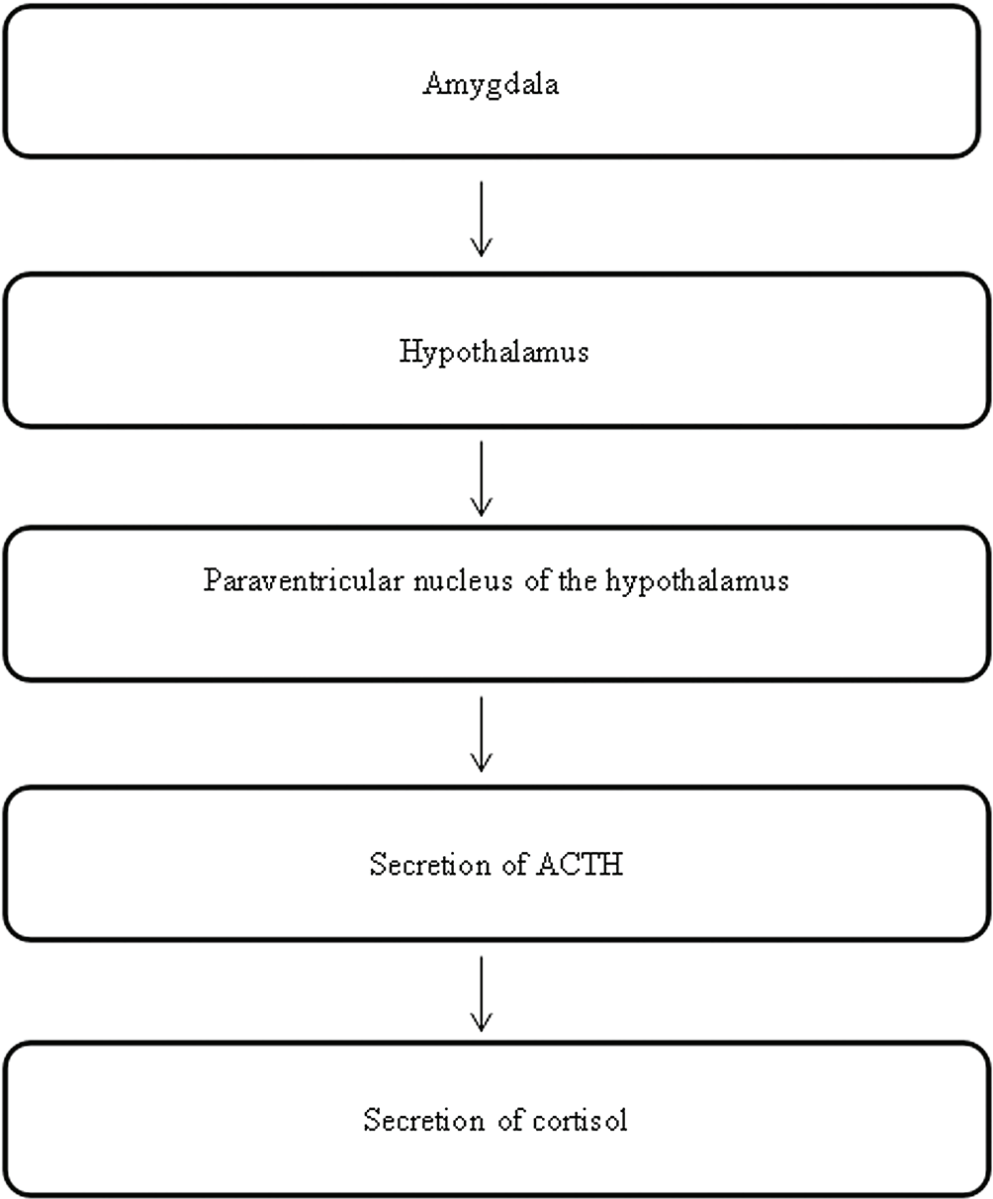
The neuropathways associated with the HPA axis.

In addition to the LC/NE system, the regulation of the HPA axis may also be implicated with depression if there is dysfunction in the liver. The hypothalamus plays a role in the physiology of depression *via* elevation in the activity of the HPA axis (Benca et al., 1992). The significance of the HPA axis in mediating physical manifestations of psychological stress has been well documented (Nestler et al., 2002; Steiger, 2007). The activity of the HPA axis is mainly related to the operation of CRH from the parvocellular neurons of the paraventricular nucleus of the hypothalamus (Steiger, 2007; Gu et al., 2018a). The secretion of CRH will stimulate the release of the adrenocorticotropic hormone (ACTH), secreting cortisol in humans and corticosterone in rats from the anterior pituitary. Most neuroendocrine studies of patients with clinical depression report elevated cortisol secretion and ACTH due to the impairment in the negative feedback system of cortisol to the HPA (Tsang and Fung, 2008) (Figure 4).

In addition, the body state named “fight or flight,” can be provided by elevated cortisol levels (Wang et al., 2017). Since the negative feedback of the HPA axis and cortisol is impaired, a higher level of HPA axis activity will lead to reduced vagal modulation or excessive activation of sympathetic neurons, resulting in physiological activation, such as increased heart rate, peripheral vasoconstriction, elevated body temperature, and increased body metabolic rate (Kales and Kales, 1984; Kales et al., 1987; Vgontzas et al., 2001; Bonnet and Arand, 2003). The above suggests that depression is closely related to over-secretion of ACTH and cortisol secretion.

### ANS Dysregulation

**Figure 5:**
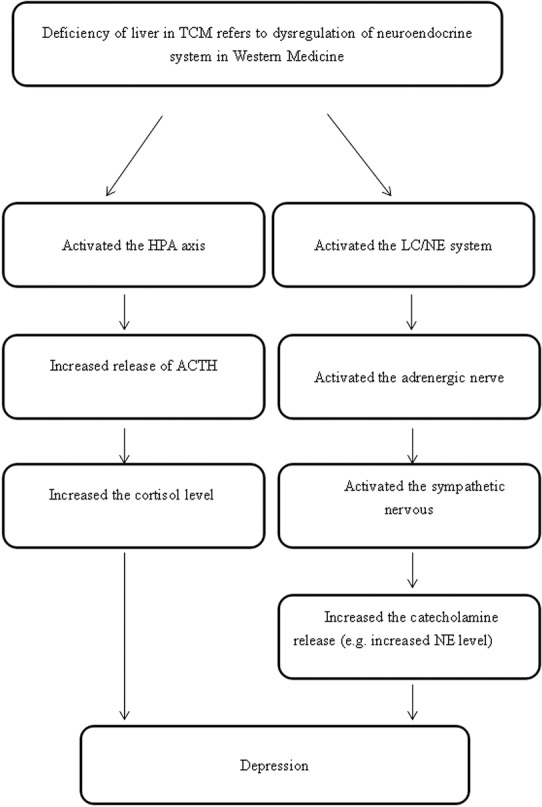
The hypothesized pathological pathways linking liver dysfunction and depression.

Apart from the function of regulating emotions in the liver, the digestive function of the spleen and stomach also depends on the movement of liver qi in TCM theory. If there is dysfunction in the liver, the digestive activities are impaired. People may exhibit the symptoms of belching, sour regurgitation, and nausea or vomiting. Lastly, the flow of bile is affected by liver function. If dysfunction of the liver occurs, the flow of bile may stagnate, leading to bitter taste in the mouth, belching, or jaundice and, resulting in sleep disturbance. An experimental study found that dysfunction of the ANS could be one of the reasons for emotional disturbance and functional dyspepsia (Vgontzas et al., 2001). Moreover, studies have shown that there is a correlation between the symptoms of stagnation of liver qi, deficiency of bile secretion, and intestinal malabsorption (Jin et al., 1985; Yue and Tian, 1995) (Figure 5).

### Tryptophan and Serotonin Deficiencies

The spleen is an abdominal organ which is involved in the production and removal of blood cells, and it is a part of the immune system according to Western medicine. However, the definition of spleen in TCM theory is different, with a broader implication than in Western medicine. It refers not only to the organ itself but also to the functions of digestion (including the pancreas and small intestine) with regard to depression (Yu, 2013).

The primary function of the spleen is to aid the stomach in the digestion of food by transporting and transforming nutrients from food and water, absorbing the nourishment, and separating the usable part of food and water from the unusable part (Giovanni, 1989). Once food and water are ingested, the spleen and stomach work closely together in digesting, extracting, and transporting the essence from food and water to the body. When the spleen is working properly, digestion will be normal and a person will have a good appetite, normal absorption, an adequate energy supply, and regular bowel movements (Giovanni, 1989). As the liver has the function of assisting the digestive functions of the spleen and stomach, if liver does not function properly, it will affect the spleen function, resulting in poor appetite, indigestion, and abdominal distension. In five-element terms, this corresponds to “Wood overacting on Earth.”

**Figure 6:**
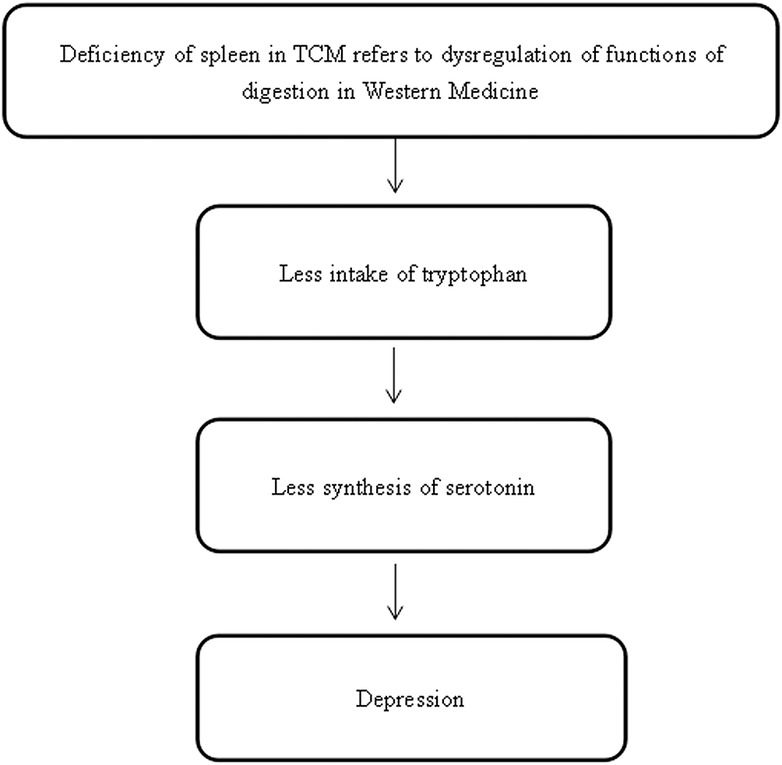
The hypothesized pathological neuropathways linking spleen dysfunction and depression.

Previous research aligns with our postulation. It has been reported that patients with dysfunction of the spleen have a low concentration of urine amylase, an insufficient concentration of serum gastrin, and a low frequency of peristalsis of the stomach (Jia et al., 1999; Tao et al., 2005; Zhang, 2006). A review showed that compared to patients suffering from only one gastrointestinal disease, patients with comorbid gastrointestinal diseases are more likely to experience anxiety, depression, and insomnia, with pathogeneses of visceral hypersensitivity, altered gastrointestinal disease motility, infection, and stressful early life events (Yue and Tian, 1995). A study by Lindgren et al. (2012) mentioned that depression was related to the symptoms of poor appetite, heartburn, diarrhea, bloating, constipation, and epigastralgia in pilots. Moreover, tryptophan, which is an indispensable amino acid, and a precursor of serotonin and melatonin, which are thought to regulate mood, is taken from food (Zhang, 2006). Intake of tryptophan has an influence on the regulation of emotional state by influencing serotonin synthesis, and this could be considered as an effective therapy for treating depression (Hartmann, 1982; Shaw et al., 2002; Lieberman, 2003). If there is a lack of food intake that is related to deficiency in tryptophan and eventually serotonin, emotional changes such as depression could happen, which parallels the findings from previous studies (Sainio et al., 1996; Birdsall, 1998; Lieberman, 2003; Le Floc’h et al., 2011; Yao et al., 2011). These studies provided preliminary evidence to support the postulation that stagnation of liver qi and spleen deficiency in depressed people in terms of TCM theory may parallel the abnormal functions of digestion in patients with depression based on the Western medical viewpoint (Figure 6).

### Hypoactivation in the Frontal Cortex

The heart is a muscular organ which pumps blood throughout the body by a circulatory system that provides oxygen and nutrients, and removes metabolic wastes. However, the function of the heart is more diversified in TCM than in Western medicine. The heart is responsible for the circulation of blood and, at the same time, the regulation of mental activities (Giovanni, 1989; Zhang, 2004; Yue et al., 2005b). According to TCM, the main functions of the heart are to govern the circulation of blood, control the blood vessels, manifest on the complexion, and store the shen, which implies consciousness, mental functions, emotion, and vitality (Ross, 1985; Giovanni, 1989; Zhang, 2004; Yue et al., 2005b).

In TCM theory, a healthy heart is essential for supplying blood to all tissues in the body. When there is a dysfunction in the heart, the circulation of blood is insufficient and the four limbs may be cold. People might exhibit the symptoms of a lower body temperature, and a white or purple complexion (Giovanni, 1989). Also, storing shen, which can be translated as “spirit” or “mind,” is a part of the heart’s functions. Shen is used to point out the whole field of the emotional, mental, and spiritual aspects of a human being. In this sense, shen not only indicates the heart, but also encompasses the emotional, mental, and spiritual phenomena of all organs. If dysfunction of the heart occurs, there is not sufficient blood to nourish the shen and a person will have difficulty in maintaining a good memory and good mental health; thus, he/she may suffer from depression. Furthermore, the heart is in charge of controlling blood vessels. The function of storing shen depends on adequate nourishment from the heart blood. Therefore, there is a mutual relationship between the function of controlling blood vessels and that of storing mind. As the blood is the root of shen, if the heart blood is sufficient, the mind will be peaceful and happy, and the pulse will become regular and normal. Conversely, if the heart blood is deficient, there is insufficient blood to root the mind, which will result in mental restlessness, depression, palpitations, and a weak or irregular pulse.

**Figure 7:**
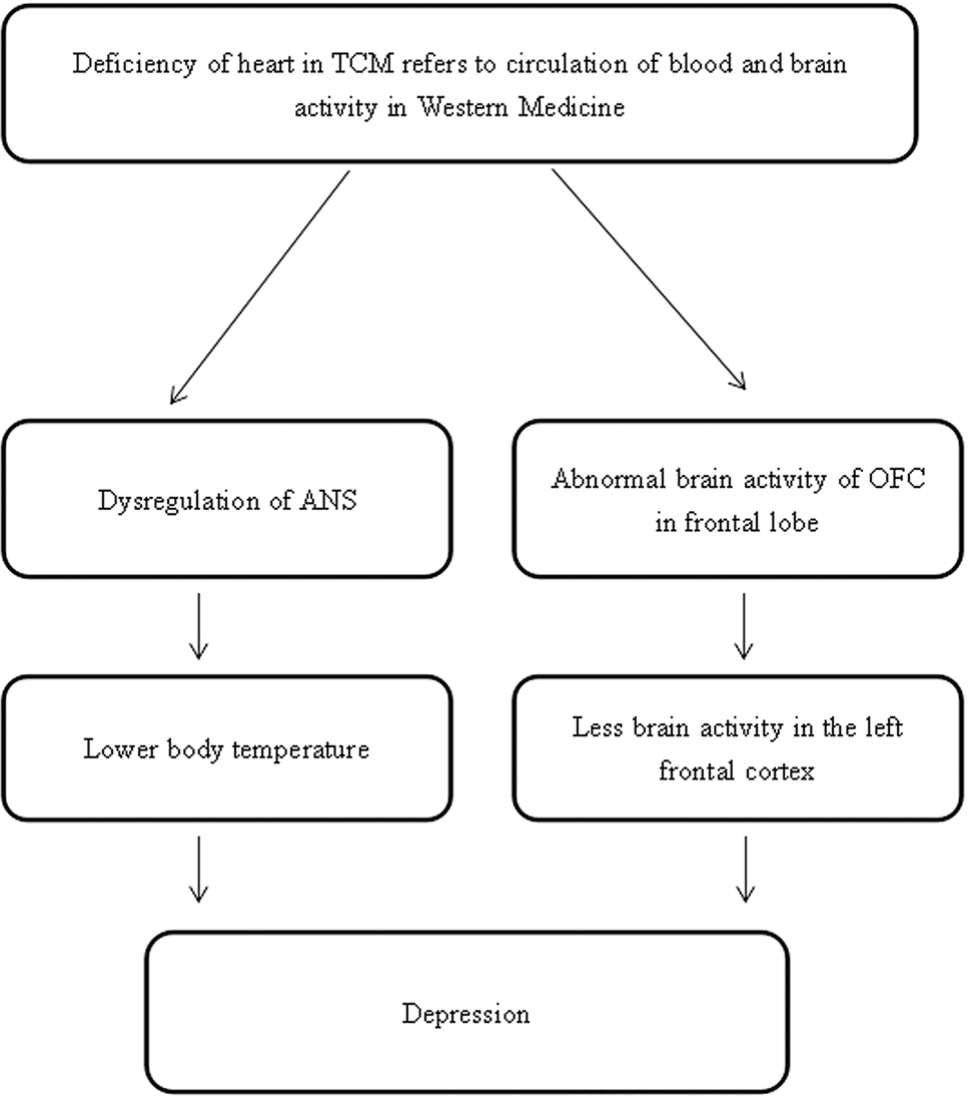
The hypothesized pathological neuropathways linking heart dysfunction and depression.

A growing number of studies support this ancient theory, showing that, compared with healthy people, patients with depression associated with deficiency of the heart and spleen have lower brain activity in the left frontal cortex region (Feng et al., 2005; Xie, 2007; Wang et al., 2008b). These findings are in line with the findings from Western medicine that major depression is related to decreased activity in the left hemisphere relative to right hemisphere, and to a decline in the activity of the left frontal cortex in people suffering from depression compared to normal people. Clinical studies reported that depression is related to altered resting-state activity in the PFC, and a growing number of findings from functional and structural imaging studies show that depression is associated with volume reduction in the left subgenual PFC region (Drevets et al., 1997; Öngür et al., 1998; Botteron et al., 2002; Wang et al., 2008a; Ye et al., 2012), because the orbitofrontal cortex (OFC) is involved in cognitive processing and decision-making, and the main function of the PFC is to extract the relevant information about a cognitive experience, so as to modulate the emotion and behavior changes (Feng et al., 2005). Moreover, studies have mentioned that the body temperature in depressed people is lower than in normal healthy people (Zhe, 2004; Lin et al., 2011); this may result from autonomic response dysfunction mediated by central adrenergic activation (Hughes et al., 2006; Hamer et al., 2007; Shinba et al., 2008) (Figure 7).

## Summary and the Way Forward

Two different systems of medicine have been used in parallel to each other for approximately 200 years. TCM is mainly based on observation and experience. In contrast, Western medicine basically relies on scientific investigation. Recent studies in Western medicine suggest that dysregulation of neurotransmitters could be one of the most vital causes of depression; while the classical texts of TCM state that dysregulation of liver qi is the main cause of depression.

Interest in the neuroscientific investigation of TCM for depression has increased dramatically in the past few decades. As the investigation of TCM using neuroscience theories and methodologies is a relatively new field of research, there are limited studies in the available literature. Knowledge of the mechanism that underlies TCM for depression is still in its infancy. However, there is emerging evidence that TCM theory might be illustrated by the changes in neurotransmitters, brain structure and function, and neuroendocrine found in people with depression.

Given the information above, we propose the following postulations linked to the liver, spleen, and heart. In terms of TCM theory, (1) liver function may be explained by the HPA axis and LC/NE system; (2) spleen function may correspond to the digestive system; and (3) heart function may refer to the circulation of the blood and the regulation of brain activity.

Further study using longitudinal study designs and larger sample sizes is recommended to advance our understanding of the mechanism of TCM for treating patients with depression. Moreover, studies applying the integrated approach of East meets West and a rigorous research design are also strongly recommended.

## Author Contributions

JY and SC searched for articles and wrote the draft version of the manuscript. HT validated the manuscript. HT, SC, WC, and JY revised the manuscript. All the authors read and approved the final manuscript.

### Conflict of Interest Statement

The authors declare that the research was conducted in the absence of any commercial or financial relationships that could be construed as a potential conflict of interest.

## References

[ref1] Abela J. R. D’Alessandro D. U. (2002). Beck’s cognitive theory of depression: a test of the diathesis-stress and causal mediation components. Br. J. Clin. Psychol. 41, 111–128. 10.1348/014466502163912, PMID: 12034000

[ref2] Aghajanian G. K. Lakoski J. M. (1984). Hyperpolarization of serotonergic neurons by serotonin and LSD: studies in brain slices showing increased K+ conductance. Brain Res. 305, 181–185. 10.1016/0006-8993(84)91137-5, PMID: 6331598

[ref3] Aldao A. Nolen-Hoeksema S. Schweizer S. (2010). Emotion-regulation strategies across psychopathology: a meta-analytic review. Clin. Psychol. Rev. 30, 217–237. 10.1016/j.cpr.2009.11.004, PMID: 20015584

[ref4] Allen B. P. (1990). Personality, social and biological perspectives on personal adjustment. (California: Pacific Grove).

[ref5] American Psychiatric Association (2013). Diagnostic and statistical manual of mental disorders: DSM-5. (Arlington, VA: American Psychiatric Association).

[ref6] Andrade L. Caraveo-anduaga J. J. Berglund P. Bijl R. V. Graaf R. D. Vollebergh W.. (2003). The epidemiology of major depressive episodes: results from the International Consortium of Psychiatric Epidemiology (ICPE) Surveys. Int. J. Methods Psychiatr. Res. 12, 3–21. 10.1002/mpr.138, PMID: 12830306 PMC6878531

[ref7] Auer D. P. Pütz B. Kraft E. Lipinski B. Schill J. Holsboer F. (2000). Reduced glutamate in the anterior cingulate cortex in depression: an in vivo proton magnetic resonance spectroscopy study. Biol. Psychiatry 47, 305–313. 10.1016/S0006-3223(99)00159-6, PMID: 10686265

[ref8] Barclay N. L. Gehrman P. R. Gregory A. M. Eaves L. J. Silberg J. L. (2015). The heritability of insomnia progression during childhood/adolescence: results from a longitudinal twin study. Sleep 38, 109–118. 10.5665/sleep.4334, PMID: 25325458 PMC4262942

[ref9] Beck A. T. (1979). Cognitive therapy of depression. (New York: Guilford Press).

[ref10] Benca R. M. Obermeyer W. H. Thisted R. A. Gillin J. C. (1992). Sleep and psychiatric disorders: a meta-analysis. Arch. Gen. Psychiatry 49, 651–668. 10.1001/archpsyc.1992.01820080059010, PMID: 1386215

[ref11] Birdsall T. C. (1998). 5-Hydroxytryptophan: a clinically-effective serotonin precursor. Altern. Med. Rev. 3, 271–280. PMID: 9727088

[ref12] Blier P. De Montigny C. (1987). Modification of 5-HT neuron properties by sustained administration of the 5-HT1A agonist gepirone: electrophysiological studies in the rat brain. Synapse 1, 470–480. 10.1002/syn.890010511, PMID: 2905533

[ref13] Blier P. Ward N. M. (2003). Is there a role for 5-HT 1A agonists in the treatment of depression? Biol. Psychiatry 53, 193–203. 10.1016/S0006-3223(02)01643-8, PMID: 12559651

[ref14] Bonnet M. Arand D. (2003). Insomnia, metabolic rate and sleep restoration. J. Intern. Med. 254, 23–31. 10.1046/j.1365-2796.2003.01176.x, PMID: 12823640

[ref15] Botteron K. N. Raichle M. E. Drevets W. C. Heath A. C. Todd R. D. (2002). Volumetric reduction in left subgenual prefrontal cortex in early onset depression. Biol. Psychiatry 51, 342–344. 10.1016/S0006-3223(01)01280-X, PMID: 11958786

[ref16] Bowden C. Theodorou A. E. Cheetham S. C. Lowther S. Katona C. L. Crompton M. R.. (1997). Dopamine D 1 and D 2 receptor binding sites in brain samples from depressed suicides and controls. Brain Res. 752, 227–233. 10.1016/S0006-8993(96)01460-6, PMID: 9106461

[ref17] Campbell-Sills L. Barlow D. H. Brown T. A. Hofmann S. G. (2006). Acceptability and suppression of negative emotion in anxiety and mood disorders. Emotion 6:587. 10.1037/1528-3542.6.4.587, PMID: 17144750

[ref18] Chen W. P. Jiang S. P. Guo Q. (2015). Brief discussion of depression theory from Zhu Danxi. Jiangsu J. Tradit. Chin. Med. 47, 12–13.

[ref20] Chen W. Z. (1990). The Chinense five-elements theory in Western medicine. (China: Xueyuan Publishing House).

[ref19] Chen W. Z. Lu Z. P. (1995). Three reasons—symptoms differentiation: (Haikou: Hainan International Press and Publication Center).

[ref21] Coppen A. Shaw D. M. Malleson A. (1965). Changes in 5-hydroxytryptophan metabolism in depression. Br. J. Psychiatry 111, 105–107. 10.1192/bjp.111.470.105, PMID: 14261721

[ref22] Copstead L. E. Banasik J. L. (2010). Pathophysiology. (St. Louis, Mo: Saunders Elsevier).

[ref23] Crabtree J. W. Lodge D. Bashir Z. I. Isaac J. T. (2013). GABAA, NMDA and mGlu2 receptors tonically regulate inhibition and excitation in the thalamic reticular nucleus. Eur. J. Neurosci. 37, 850–859. 10.1111/ejn.12098, PMID: 23294136 PMC4243027

[ref24] Drevets W. C. Bogers W. Raichle M. E. (2002). Functional anatomical correlates of antidepressant drug treatment assessed using PET measures of regional glucose metabolism. Eur. Neuropsychopharmacol. 12, 527–544. 10.1016/S0924-977X(02)00102-5, PMID: 12468016

[ref25] Drevets W. C. Price J. L. Simpson J. R. Jr. Todd R. D. Reich T. Vannier M.. (1997). Subgenual prefrontal cortex abnormalities in mood disorders. Nature 386, 824. 10.1038/386824a0, PMID: 9126739

[ref26] Feng Z. H. Wang K. Wang C. Q. Meng Y. Yi S. J. (2005). The neural basis of emotional cognition. Chin. J. Neurol. 38, 525–527. 10.1016/j.nicl.2018.05.009

[ref27] Ferrari A. J. Charlson F. J. Norman R. E. Patten S. B. Freedman G. Murray C. J.. (2013). Burden of depressive disorders by country, sex, age, and year: findings from the global burden of disease study 2010. PLoS Med. 10:e1001547. 10.1371/journal.pmed.1001547, PMID: 24223526 PMC3818162

[ref28] Gao X. Sun P. Qiao M. Wei S. Xue L. Zhang H. (2014). Shu-Yu capsule, a Traditional Chinese Medicine formulation, attenuates premenstrual syndrome depression induced by chronic stress constraint. Mol. Med. Rep. 10, 2942–2948. 10.3892/mmr.2014.2599, PMID: 25270424

[ref29] General Office of the State Administration of Traditional Chinese Medicine and School of Management of Beijing University of Chinese Medicine (2006). China statistical yearbook of Chinese medicine. (China: China Academic Journals Electronic Publishing House).

[ref30] General U. S. P. H. S. O. O. T. S., Services, C.f.M.H., Abuse, U.S.S., Administration, M.H.S., and Health, N.I.o.M (2001). Mental health: Culture, race, and ethnicity: A supplement to mental health: A report of the Surgeon General. (Rockville: Department of Health and Human Services, US Public Health Service). 20669516

[ref31] Giovanni M. (1989). The Foundations of Chinese Medicine: A comprehensive text for acupuncturists and herbalists. (Edinburgh, UK: Churchill Livingstone), 219–268.

[ref32] Glennon R. Dukat M. (1991). Serotonin receptors and their ligands: A lack of selective agents. Pharmacol. Biochem. Be 40, 1009–1017. 10.1016/0091-3057(91)90121-H 1816555

[ref33] Gratz K. L. Roemer L. (2004). Multidimensional assessment of emotion regulation and dysregulation: development, factor structure, and initial validation of the difficulties in emotion regulation scale. J. Psychopathol. Behav. Assess. 26, 41–54. 10.1023/B:JOBA.0000007455.08539.94

[ref34] Gu S. Gao M. Yan Y. Wang F. Tang Y.-Y. Huang J. H. (2018a). The neural mechanism underlying cognitive and emotional processes in creativity. Front. Psychol. 9:1924. 30429805 10.3389/fpsyg.2018.01924 PMC6220028

[ref35] Gu S. Wang W. Wang F. Huang J. H. (2016). Neuromodulator and emotion biomarker for stress induced mental disorders. Neural Plast. 2016:2609128. 10.1155/2016/2609128, PMID: 27051536 PMC4808661

[ref36] Gu S. M. Jing L. Y. Goa M. D. Wang F. S. (2018b). Neuropsychological perspective of TCM emotions theory. Mod. Trad. Chin. Med. Materia Medica 20, 173–182.

[ref37] Guo Y. M. Liu C. F. (2002). Wang Yanheng’s experience in the treatment of depression. Hebei Chin. Med. 24, 100–101.

[ref38] Hamer M. Tanaka G. Okamura H. Tsuda A. Steptoe A. (2007). The effects of depressive symptoms on cardiovascular and catecholamine responses to the induction of depressive mood. Biol. Psychol. 74, 20–25. 10.1016/j.biopsycho.2006.06.003, PMID: 16860921

[ref39] Hartmann E. (1982). Effects of L-tryptophan on sleepiness and on sleep. J. Psychiatr. Res. 17, 107–113. 10.1016/0022-3956(82)90012-7, PMID: 6764927

[ref40] Hasler G. van der Veen J. W. Tumonis T. Meyers N. Shen J. Drevets W. C. (2007). Reduced prefrontal glutamate/glutamine and γ-aminobutyric acid levels in major depression determined using proton magnetic resonance spectroscopy. Arch. Gen. Psychiatry 64, 193–200. 10.1001/archpsyc.64.2.193, PMID: 17283286

[ref41] Heath A. C. Todorov A. A. Nelson E. C. Madden P. A. Bucholz K. K. Martin N. G. (2002). Gene–environment interaction effects on behavioral variation and risk of complex disorders: the example of alcoholism and other psychiatric disorders. Twin Res. Hum. Genet. 5, 30–37. 10.1375/twin.5.1.30, PMID: 11893279

[ref42] Hoyer D. Pazos A. Probst A. Palacios J. (1986). Serotonin receptors in the human brain. II. Characterization and autoradiographic localization of 5-HT 1C and 5-HT 2 recognition sites. Brain Res. 376, 97–107. 10.1016/0006-8993(86)90903-0, PMID: 2941113

[ref43] Hughes J. W. Casey E. Luyster F. Doe V. H. Waechter D. Rosneck J. (2006). Depression symptoms predict heart rate recovery after treadmill stress testing. Am. Heart J. 151, 1122. e1–1122. e6. 10.1016/j.ahj.2006.02.004 16644348

[ref44] Ikram H. Haleem D. J. (2017). Repeated treatment with reserpine as a progressive animal model of depression. Pak. J. Pharm. Sci. 30, 897–902. PMID: 28653936

[ref45] Jia J. Zhu Z. Q. Zhang L. (1999). The mechanism of spleen and stomach dysfunction. J. Shenyang Phys. Univ. 39–41.

[ref46] Jin G. L. Liang Y. (1997). Seasonal pathogenesis of depression and its enlightenment. J. Beijing Univ. Tradit. Chin. Med. 20, 15–16.

[ref47] Jin Y. Q. (2000). Modern research and clinical practice of liver in traditional Chinese Medicine. (Beijing: People’s Medical Publishing House).

[ref48] Jin Y. Q. Liu H. Y. Li X. W. (1985). Analysis of intestinal absorption dysfunction in 227 patients wsith stagnation of liver qi and spleen deficiency. J. Hunan Med. Univ. 10, 38–39.

[ref50] Kales A. Soldatos C. R. Kales J. D. (1987). Sleep disorders: insomnia, sleepwalking, night terrors, nightmares, and enuresis. Ann. Intern. Med. 106, 582–592. 10.7326/0003-4819-106-4-582, PMID: 3548525

[ref49] Kales J. D. Kales A. (1984). Evaluation and treatment of insomnia. Ann. Intern. Med. 101:886. 10.7326/0003-4819-101-6-886_1

[ref51] Keji C. Hao X. (2003). The integration of traditional Chinese medicine and Western medicine. Eur. Rev. 11, 225–235. 10.1017/S106279870300022X

[ref52] Kessler R. C. Berglund P. Demler O. Jin R. Koretz D. Merikangas K. R.. (2003). The epidemiology of major depressive disorder: results from the National Comorbidity Survey Replication (NCS-R). JAMA 289, 3095–3105. 10.1001/jama.289.23.3095, PMID: 12813115

[ref53] Kring A. M. Werner K. H. (2004). “Emotion regulation and psychopathology” in The regulation of emotion. eds. Philippot P. Feldman R. S. (Mahwah, NJ, US: Lawrence Erlbaum Associates Publishers), 359–385.

[ref54] Krystal J. H. Sanacora G. Blumberg H. Anand A. Charney D. Marek G.. (2002). Glutamate and GABA systems as targets for novel antidepressant and mood-stabilizing treatments. Mol. Psychiatry 7, S71–S80. 10.1038/sj.mp.4001021, PMID: 11986998

[ref55] Le Floc’h N. Otten W. Merlot E. (2011). Tryptophan metabolism, from nutrition to potential therapeutic applications. Amino Acids 41, 1195–1205. 10.1007/s00726-010-0752-7, PMID: 20872026

[ref56] Li J. B. Wang Y. H. (1985). Characteristics of autonomic dysfunction in patients with liver depression and spleen deficiency syndrome. J. Hunan Med. Univ. 1:018.

[ref57] Lieberman H. R. (2003). Nutrition, brain function and cognitive performance. Appetite 40, 245–254. 10.1016/S0195-6663(03)00010-2, PMID: 12798782

[ref58] Lin H. P. Lin H. Y. Lin W. L. Huang A. C. W. (2011). Effects of stress, depression, and their interaction on heart rate, skin conductance, finger temperature, and respiratory rate: sympathetic-parasympathetic hypothesis of stress and depression. J. Clin. Psychol. 67, 1080–1091. 10.1002/jclp.20833, PMID: 21905026

[ref59] Lindgren T. Runeson R. Wahlstedt K. Wieslander G. Dammström B.-G. Norbäck D. (2012). Digestive functional symptoms among commercial pilots in relation to diet, insomnia, and lifestyle factors. Aviat. Space Environ. Med. 83, 872–878. 10.3357/ASEM.3309.2012, PMID: 22946351

[ref60] Liu B. L. (1991). Experience of Jieyu Decoction in the treatment of 31 cases of latent depression. Tianjin Tradit. Chin. Med. 8:326.

[ref61] Liu C.-C. Wu Y.-F. Feng G.-M. Gao X.-X. Zhou Y.-Z. Hou W.-J.. (2015). Plasma-metabolite-biomarkers for the therapeutic response in depressed patients by the traditional Chinese medicine formula Xiaoyaosan: a 1 H NMR-based metabolomics approach. J. Affect. Disord. 185, 156–163. 10.1016/j.jad.2015.05.005, PMID: 26186531

[ref62] Martini F. (2015). Fundamentals of anatomy & physiology. (Boston: Pearson).

[ref63] Molander L. Randrup A. (1976). Effects of thymoleptics on behavior associated with changes in brain dopamine. Psychopharmacology 49, 139–144. 10.1007/BF00427282, PMID: 825900

[ref64] Moret C. Briley M. (2011). The importance of norepinephrine in depression. Neuropsychiatr. Dis. Treat. 7, 9–13. 10.2147/NDT.S19619, PMID: 21750623 PMC3131098

[ref65] Nestler E. J. Barrot M. DiLeone R. J. Eisch A. J. Gold S. J. Monteggia L. M. (2002). Neurobiology of depression. Neuron 34, 13–25. 10.1016/S0896-6273(02)00653-0, PMID: 11931738

[ref66] Ng S.-M. Chan C. L. Ho D. Y. Wong Y.-Y. Ho R. T. (2006). Stagnation as a distinct clinical syndrome: comparing ‘Yu’(stagnation) in traditional Chinese medicine with depression. Br. J. Soc. Work 36, 467–484. 10.1093/bjsw/bcl008

[ref67] Nyberg S. Farde L. Eriksson L. Halldin C. Eriksson B. (1993). 5-HT 2 and D 2 dopamine receptor occupancy in the living human brain. Psychopharmacology 110, 265–272. 10.1007/BF02251280, PMID: 7530376

[ref68] Öngür D. Drevets W. C. Price J. L. (1998). Glial reduction in the subgenual prefrontal cortex in mood disorders. Proc. Natl. Acad. Sci. 95, 13290–13295. 9789081 10.1073/pnas.95.22.13290 PMC23786

[ref69] Ou M. (1988). Chinese-English dictionary of traditional Chinese medicine. (Hong Kong: Joint Publishing (Hong Kong) Co Ltd.).

[ref70] Pandey G. N. (1997). Altered serotonin function in suicide. Ann. N. Y. Acad. Sci. 836, 182–201. 10.1111/j.1749-6632.1997.tb52360.x, PMID: 9616799

[ref71] Park J. (2002). Acupuncture in the treatment of depression: a manual for practice and research. Focus. Altern. Complement. Ther. 7:69. 10.1111/j.2042-7166.2002.tb03344.x

[ref72] Ross J. (1985). Zang Fu, the organ systems of traditional Chinese medicine: Functions, interrelationships and patterns of disharmony in theory and practice. (Churchill Livingstone: Elsevier Health Sciences).

[ref73] Sainio E.-L. Pulkki K. Young S. (1996). L-Tryptophan: biochemical, nutritional and pharmacological aspects. Amino Acids 10, 21–47. 10.1007/BF00806091, PMID: 24178430

[ref74] Samson J. A. Mirin S. M. Griffin M. Borrelli D. Schildkraut J. J. (1994). Urinary MHPG and clinical symptoms in patients with unipolar depression. Psychiatry Res. 51, 157–165. 10.1016/0165-1781(94)90035-3, PMID: 8022950

[ref75] Sanacora G. Mason G. F. Rothman D. L. Behar K. L. Hyder F. Petroff O. A.. (1999). Reduced cortical γ-aminobutyric acid levels in depressed patients determined by proton magnetic resonance spectroscopy. Arch. Gen. Psychiatry 56, 1043–1047. 10.1001/archpsyc.56.11.1043, PMID: 10565505

[ref76] Scheid V. (2013). Depression, constraint, and the liver: (dis) assembling the treatment of emotion-related disorders in Chinese medicine. Cult. Med. Psychiatry 37, 30–58. 10.1007/s11013-012-9290-y, PMID: 23315392 PMC3586067

[ref77] Schildkraut J. J. (1965). The catecholamine hypothesis of affective disorders: a review of supporting evidence. Am. J. Psychiatr. 122, 509–522. 10.1176/ajp.122.5.509, PMID: 5319766

[ref78] Shanahan M. J. Hofer S. M. (2005). Social context in gene–environment interactions: retrospect and prospect. J. Gerontol. Ser. B Psychol. Sci. Soc. Sci. 60, 65–76. 10.1093/geronb/60.Special_Issue_1.65 15863711

[ref79] Shaw K. A. Turner J. Del Mar C. (2002). Tryptophan and 5-Hydroxytryptophan for depression. Cochrane Database Syst. Rev. 1:CD003198. 10.1002/14651858.CD003198 11869656

[ref80] Shinba T. Kariya N. Matsui Y. Ozawa N. Matsuda Y. Yamamoto K.i. (2008). Decrease in heart rate variability response to task is related to anxiety and depressiveness in normal subjects. Psychiatry Clin. Neurosci. 62, 603–609. 10.1111/j.1440-1819.2008.01855.x, PMID: 18950382

[ref81] Smith S. M. Vale W. W. (2006). The role of the hypothalamic-pituitary-adrenal axis in neuroendocrine responses to stress. Dialogues Clin. Neurosci. 8, 383–395. PMID: 17290797 10.31887/DCNS.2006.8.4/ssmith PMC3181830

[ref82] So R. W. L. Wong H. S. Ko K. M. (2015). A traditional Chinese medicine approach in treating depression by promoting liver qi circulation: a western medicine perspective. Chin. Med. 6, 187–195. 10.4236/cm.2015.64021

[ref83] Soygüt G. Savaşir I. (2001). The relationship between interpersonal schemas and depressive symptomatology. J. Couns. Psychol. 48, 359–364. 10.1037/0022-0167.48.3.359

[ref84] Spiegelhalder K. Fuchs L. Ladwig J. Kyle S. D. Nissen C. Voderholzer U.. (2011). Heart rate and heart rate variability in subjectively reported insomnia. J. Sleep Res. 20, 137–145. 10.1111/j.1365-2869.2010.00863.x, PMID: 20626615

[ref85] Steiger A. (2007). Neurochemical regulation of sleep. J. Psychiatr. Res. 41, 537–552. 10.1016/j.jpsychires.2006.04.007, PMID: 16777143

[ref86] Sullivan P. F. Neale M. C. Kendler K. S. (2000). Genetic epidemiology of major depression: review and meta-analysis. Am. J. Psychiatr. 157, 1552–1562. 10.1176/appi.ajp.157.10.1552, PMID: 11007705

[ref88] Tao L. Zhang M. S. Wang H. B. Li W. (2005). A Clinical study on Jian Pi Li Qi Huo Xue decoction in the treatment of functional dyspepsia. Chin. J. Inf. Tradit. Chin. Med. 12, 11–13.

[ref89] Ted J. K. (2000). The web that has no weaver: Understanding Chinese medicine. (Chicago, Ill, USA: McGraw-Hill).

[ref90] Tian P. (2011). Convergence: where west meets east. Nature 480, S84–S86. 10.1038/480S84a, PMID: 22190086

[ref91] Tsang H. W. Fung K. M. (2008). A review on neurobiological and psychological mechanisms underlying the anti-depressive effect of qigong exercise. J. Health Psychol. 13, 857–863. 10.1177/1359105308095057, PMID: 18809635

[ref92] Veith I. (2015). The yellow emperor’s classic of internal medicine. (Oakland, CA: University of California Press).

[ref93] Verbeeck W. Berk M. Paiker J. Jersky B. (2001). The prolactin response to sulpiride in major depression: the role of the D 2 receptor in depression. Eur. Neuropsychopharmacol. 11, 215–220. 10.1016/S0924-977X(01)00086-4, PMID: 11418281

[ref94] Vgontzas A. N. Bixler E. O. Lin H.-M. Prolo P. Mastorakos G. Vela-Bueno A.. (2001). Chronic insomnia is associated with nyctohemeral activation of the hypothalamic-pituitary-adrenal axis: clinical implications. J. Clin. Endocrinol. Metabol. 86, 3787–3794. 10.1210/jcem.86.8.7778, PMID: 11502812

[ref95] Wägner A. Montero D. Mårtensson B. Siwers B. Åsberg M. (1990). Effects of fluoxetine treatment of platelet 3 H-imipramine binding, 5-HT uptake and 5-HT content in major depressive disorder. J. Affect. Disord. 20, 101–113. 10.1016/0165-0327(90)90123-P, PMID: 2176228

[ref96] Wang F. Pan F. Shapiro L. A. Huang J. H. (2017). Stress induced neuroplasticity and mental disorders. Neural Plast. 2017:9634501. 10.1155/2017/9634501, PMID: 28785487 PMC5530430

[ref97] Wang L. LaBar K. S. Smoski M. Rosenthal M. Z. Dolcos F. Lynch T. R. (2008a). Prefrontal mechanisms for executive control over emotional distraction are altered in major depression. Psychiatry Res. Neuroimaging 163, 143–155. 10.1016/j.pscychresns.2007.10.004 PMC2553159 18455373

[ref98] Wang Y. Lu Z. (2002). Internal medicine of traditional Chinese medicine. (Shanghai: Publishing House of Shanghai University of Traditional Chinese Medicine 74).

[ref119] Wang Y. Yao W. (2002). Internal medicine of traditional Chinese medicine. (Shanghai: Shanghai University of Traditional Chinese Medicine).

[ref99] Wang Y. L. Qin S. L. Guo R. J. Teng J. Du Y. W. Jiang S. Y. (2008b). A comparative study of nonlinear analysis of EEG in patients with depression 26, 1845–1848.

[ref100] Wei S. Hou J. L. Chao Y. B. Du X. Y. Zong S. B. (2012). The analysis of serum monoamine neurotransmitters in rats with premenstrual syndrome and liver qi stagnation. Chin. J. Integr. Med. 10, 925–930. 10.3736/jcim20120814 22883410

[ref101] Wesolowska A. (2002). In the search for selective ligands of 5-HT5, 5-HT6 and 5-HT7 serotonin receptors. Pol. J. Pharmacol. 54, 327–341. PMID: 12523486

[ref102] Willner P. (1983). Dopamine and depression: a review of recent evidence. I. Empirical studies. Brain Res. Rev. 6, 211–224. 10.1016/0165-0173(83)90005-X 6140979

[ref87] Wu P. Sun X. Y. Sun F. Y. (1963). Annotated Shen Nong’s Herbal. (Beijing: People’s Medical Publishing House (PMPH)).

[ref103] Xie L. J. (2007). A clinical study on the treatment of depression with the method of tonifying kidney and regulating qi. (Beijing University of Chinese Medicine).

[ref104] Yan C. Xu Z. W. (2005). To investigate the central nerve mechanism of liver regulating emotion function. Chin. J. Integr. Tradit. West. Med. 25, 459–462. 15957846

[ref105] Yao K. Fang J. Yin Y. Feng Z.-M. Tang Z.-R. Wu G. (2011). Tryptophan metabolism in animals: important roles in nutrition and health. Front. Biosci. 3, 286–297. 10.2741/s152 21196377

[ref106] Ye T. Peng J. Nie B. Gao J. Liu J. Li Y.. (2012). Altered functional connectivity of the dorsolateral prefrontal cortex in first-episode patients with major depressive disorder. Eur. J. Radiol. 81, 4035–4040. 10.1016/j.ejrad.2011.04.058, PMID: 22939367

[ref107] Young S. N. (2007). How to increase serotonin in the human brain without drugs. J. Psychiatry Neurosci. 32, 394–399. PMID: 18043762 PMC2077351

[ref108] Yu Q. L. (2013). Redescription of Zang Spleen Model in Modern Anatomico-functional Terms. J. Chin. Med. 24, 183–209. 10.3966/101764462013122402001

[ref109] Yuan Y. G. Zhang X. B. Zhang S. N. (2004). A comparative study of plasma monoamine neurotransmitter concentrations in patients with depression and anxiety disorder. Chin. J. Behav. Med. Sci. 13, 30–31.

[ref110] Yue G. X. Chen J. X. Wang Z. F. (2005a). Physiological basis of Liver in traditional Chinese medicine. J. Beijing Univ. Tradit. Chin. Med. 28, 1–4.

[ref111] Yue G. X. Chen J. X. Wang Z. F. (2005b). The role of heart, kidney and liver in stress reaction based on TCM Liaoning. J. Tradit. Chin. Med. 32, 528–530.

[ref112] Yue W. H. Tian X. M. (1995). The mechanism of anger and its damage of liver. J. Med. Philos. 16, 481–483.

[ref113] Zhang C. Z. Deng T. T. Lai C. (2011). Confucian filiality. (Beijing: Chinese Medical Science and Technology Press).

[ref114] Zhang G. X. (2004). Analysis of TCM thermoregulation mechanism. Fujian Tradit. Chin. Med. 35, 42–43.

[ref115] Zhang Y. (2006). A study on clinical manifestations of spleen deficiency differentiation. (Guangzhou: Guangzhou University of Chinese Medicine).

[ref116] Zhao J. P. (1992). Jieyu decoction for depression. J. Sichuan Tradit. Chin. Med. 8:031.

[ref117] Zhao Z. S. Zhao M. (1999). Clinical observation on 180 cases of depression treated with Kang Wei Kang. J. Shandong Tradit. Chin. Med. 18, 110–111.

[ref118] Zhe L. (2004). The prescriptions of prescriptions for antidepressant medicine in traditional Chinese medicine. (Nanjin: Nanjing University of Traditional Chinese Medicine).

[ref120] Zorumski C. F. Paul S. M. Izumi Y. Covey D. F. Mennerick S. (2013). Neurosteroids, stress and depression: potential therapeutic opportunities. Neurosci. Biobehav. Rev. 37, 109–122. 10.1016/j.neubiorev.2012.10.005, PMID: 23085210 PMC3591791

